# Phase II study of bevacizumab, cisplatin, and docetaxel plus maintenance bevacizumab as first-line treatment for patients with advanced non-squamous non-small-cell lung cancer combined with exploratory analysis of circulating endothelial cells: Thoracic Oncology Research Group (TORG)1016

**DOI:** 10.1186/s12885-018-4150-y

**Published:** 2018-03-02

**Authors:** Satoshi Ikeda, Terufumi Kato, Takashi Ogura, Akimasa Sekine, Tsuneyuki Oda, Noriyuki Masuda, Satoshi Igawa, Ken Katono, Sakiko Otani, Kouzo Yamada, Haruhiro Saito, Tetsuro Kondo, Yukio Hosomi, Yoshiro Nakahara, Masanori Nishikawa, Keiko Utumi, Yuki Misumi, Takeharu Yamanaka, Kentaro Sakamaki, Hiroaki Okamoto

**Affiliations:** 1 Department of Respiratory Medicine, Kanagawa Cardiovascular and Respiratory Center, Tomioka-Higashi 6-16-1, Kanazawa-ku, Yokohama, Japan; 2 Department of Thoracic Oncology, Kanagawa Cancer Center, Yokohama, Japan; 3 Department of Respiratory Medicine, Kitasato University Hospital, Sagamihara, Japan; 4 Division of Thoracic Oncology and Respiratory Medicine, Tokyo Metropolitan Cancer and Infectious Diseases Center Komagome Hospital, Tokyo, Japan; 5 Department of Respiratory Medicine, Fujisawa City Hospital, Fujisawa, Japan; 6 Department of Respiratory Medicine, Ibaraki Prefectural Central Hospital, Kasama, Japan; 7 Department of Respiratory Medicine and Medical Oncology, Yokohama Municipal Citizen’s Hospital, Yokohama, Japan; 8 Department of Biostatistics, Yokohama City University School of Medicine, Yokohama, Japan

**Keywords:** Non-small cell lung cancer, Docetaxel, Bevacizumab, Circulating endothelial cell

## Abstract

**Background:**

Preclinical studies have demonstrated that docetaxel and bevacizumab may act synergistically by decreasing endothelial cell proliferation and preventing circulating endothelial progenitor mobilization. The objective of this study was to assess the efficacy and safety of a combination therapy of bevacizumab, cisplatin, and docetaxel in chemotherapy-naive Japanese patients with advanced non-squamous non-small-cell lung cancer (NSCLC).

**Methods:**

Eligible patients were chemotherapy-naive and had advanced/recurrent non-squamous NSCLC. The patients received 4 cycles of docetaxel (60 mg/m^2^), cisplatin (80 mg/m^2^), and bevacizumab (15 mg/kg) once every 3 weeks, followed by bevacizumab as maintenance therapy, every 3 weeks until disease progression or attainment of unacceptable toxicity level. The primary endpoint was objective response rate (ORR). The numbers of circulating endothelial cells (CEC) were also estimated on days 1 and 8 of the first cycle for the exploratory analysis of efficacy prediction.

**Results:**

A total of 47 patients were enrolled from October 2010 to April 2012. Bevacizumab as maintenance therapy was administered to 41 patients (87.2%), and the median number of total treatment cycles was 9 (range: 1–36). ORR, median progression-free survival (PFS), and median overall survival of the patients were 74.5%, 9.0 months, and 27.5 months, respectively. The most common grade 3/4 adverse event was neutropenia (95.7%), followed by leukopenia (59.6%) and hypertension (46.8%). PFS was longer in patients with ≥10 count increase in CECs than that in patients with < 10 count increase in CECs (respective median PFS of 11.0 months versus 6.90 months) although the difference was not statistically significant (*p = 0.074*).

**Conclusions:**

A combination therapy of bevacizumab, cisplatin, and docetaxel, followed by bevacizumab as maintenance was highly effective in patients with non-squamous NSCLC despite the high incidence of grade 3/4 neutropenia. The increase in CEC count between days 1 and 8 may predict the efficacy of our bevacizumab-contained treatment regimen.

**Trial registration:**

UMIN Clinical Trial Registry; UMIN000004368. Registered date; October 11, 2010 (Retrospectively registered).

**Electronic supplementary material:**

The online version of this article (10.1186/s12885-018-4150-y) contains supplementary material, which is available to authorized users.

## Background

Among the platinum-based doublet chemotherapy for patients with advanced non-small-cell lung cancer (NSCLC), docetaxel is one of the best taxane composition combined with cisplatin [1]. The cytotoxic activity of docetaxel is mainly exerted by promoting the assembly of microtubules from tubulin dimers, which in turn, inhibits the de-polymerization of tubulin that stabilizes microtubules in the cells [2–4]. This results in the inhibition of DNA, RNA, and protein synthesis. In addition, docetaxel inhibits vascular endothelial growth factor (VEGF)-induced neovascularization in vivo and has an anti-angiogenic effect [5, 6]. Furthermore, the blockade of the VEGF pathway has emerged as a rational target for therapeutic intervention owing to the dependence of tumor survival and growth on angiogenesis. In recent phase III studies, Bevacizumab, a humanized monoclonal antibody that inhibits VEGF, in combination with platinum-based chemotherapy has been reported to increase both objective response rates (ORRs) and progression-free survival (PFS) in chemotherapy-naive patients with advanced NSCLC [7, 8]. Although carboplatin/paclitaxel has often been selected along with bevacizumab, preclinical studies have revealed that docetaxel decreases endothelial cell proliferation, thereby increasing the efficacy of VEGF receptor blockade by bevacizumab [9]. In addition, bevacizumab prevents the mobilization of circulating endothelial progenitors, which are induced from the bone marrow by vascular disrupting agents, such as docetaxel, and contributes to tumor angiogenesis and growth [10]. To date, two single-arm phase II studies of bevacizumab (15 mg/kg), docetaxel (75 mg/m^2^), and cisplatin as the first-line treatment for patients with metastatic non-squamous NSCLC have revealed an acceptable toxicity profile and promising anti-tumor effect [11, 12]. However, the Japanese population appears to be more susceptible to the toxicity of docetaxel, and the approved docetaxel dose for NSCLC is 60 mg/m^2^ in Japan [13]. The efficacy and safety of 60 mg/m^2^ of docetaxel combined with bevacizumab and cisplatin in Japanese patients should be confirmed. Furthermore, it is critically important to establish biomarkers that can identify subgroups of patients who can benefit from bevacizumab for the improvement of clinical outcome and treatment costs. The change in the number of circulating endothelial cells (CECs) before cancer treatment and that after the administration of drugs has been reported to be a potential biomarker for the prediction of response to bevacizumab [14].

The objective of this study was to assess the efficacy and safety of bevacizumab, cisplatin, and docetaxel in a combination treatment regimen for chemotherapy-naive Japanese patients with non-squamous NSCLC. In addition, CECs were evaluated for the exploratory analysis of efficacy prediction.

## Methods

### Study design

This multi-center, single-arm, phase II study was conducted in accordance with the Declaration of Helsinki and the Ethical Guidelines for Clinical Research issued by the Japanese Ministry of Health, Labour and Welfare. The anticipated trial start date was October 1, 2010, and the data cutoff date was October 1, 2014. The protocol was approved by the Clinical Trial Review Committee of the Thoracic Oncology Research Group (TORG) and the Institutional Review Board or Ethics Committee of each participating center. All patients provided written informed consent. This study adheres to CONSORT guidelines.

### Study participants

The inclusions criteria for the study subjects were as follows: (1) pathologically or cytologically confirmed NSCLC; (2) stage IIIA/IIIB/IV unsuitable for curative radiotherapy or post-operative recurrent disease; (3) age ≥ 20 years and ≤75 years; (4) Eastern Cooperative Oncology Group (ECOG) performance status (PS) of 0 or 1; (5) at least one measurable lesion meeting the Response Evaluation Criteria (RECIST) (version 1.1); (6) no prior treatment except for surgery, epidermal growth factor receptor tyrosine kinase inhibitor (EGFR-TKI), and palliative radiotherapy; (7) adequate organ function (white blood cell count ≥4000 cells/μL, neutrophil count ≥2000 cells/μL, hemoglobin ≥9.0 g/dL, platelet count ≥100,000 cells/μL, aspartate aminotransferase (AST) and alanine aminotransferase (ALT) levels ≤100 IL/L, total bilirubin level ≤ 1.5 mg/dL, serum creatinine level ≤ 1.2 mg/dL, creatinine clearance level ≥ 60 mL, oxygen saturation by pulse oximetry ≥93%, proteinuria ≤1+) (8) life expectancy > 3 months; (9) adequate interval after prior treatments (2 weeks from radiotherapy, 8 weeks from lobectomy, 4 weeks from exploratory thoracotomy, and 2 weeks from pleural drainage); and (10) written informed consent. The exclusion criteria for the study subjects were as follows: (1) presence of brain metastases, (2) history of hemoptysis, (3) severe or uncontrollable comorbidities, (4) massive pleural/pericardial effusion or ascites, (5) concomitant malignancy, (6) history of peptic ulcer within the past year, or (7) regular use of anticoagulants (≤325 mg/day of aspirin was permitted).

### Treatment

The patients received 4 cycles of docetaxel (60 mg/m^2^, intravenously administered over a period of 1 h), cisplatin (80 mg/m^2^, intravenously administered over a period of 2 h), and bevacizumab (15 mg/kg, intravenously administered over a period of 1.5 h) once every 3 weeks, followed by bevacizumab alone as maintenance therapy every 3 weeks until disease progression or the attainment of unacceptable toxicity.

### Evaluation

Tumor response was evaluated by the Extramural Central Review Committee according to the RECIST. PFS was assessed from the date of enrollment to the development of the earliest sign of disease progression, as determined by chest computed tomography (CT) or magnetic resonance imaging (MRI) according to RECIST criteria, or death from any cause. Overall survival (OS) was assessed from the date of enrollment until death from any cause. Safety was assessed according to the National Cancer Institute Common Terminology Criteria for Adverse Events (CTCAE; version 3.0). Disease status was assessed by a chest CT or MRI every 6 weeks. After the confirmation of partial response (PR), the patients underwent a chest CT or MRI every 4 weeks until disease progression.

### Statistical analysis

The primary end point of the study was ORR. Based on the Simon’s two-staged design, the planned sample size of 47 patients was determined appropriate to reject a null ORR of 35% at one-sided significance level of 0.05 under an expected ORR of 55% with a power of 0.80. The secondary end points included OS, PFS, and safety. All patients were followed-up until October 1, 2014. Cumulative survival probabilities were estimated using the Kaplan–Meier method. A log-rank test was performed to compare survival among the patient groups. A *p* < 0.05 was considered statistically significant. Statistical analyses were performed using SAS Version 9.3.1.

### Analysis of CECs

The CEC counts were determined on days 1 and 8 of the first cycle for the exploratory analysis of efficacy prediction using the CellSearch® system. This system is composed of a combination of semiautomatic isolation of CECs and microscopic visualization based on immunophenotypical and morphological definitions. The blood sample (10 ml) is first collected into a CellSave™ tube, which contains a solution of Na2EDTA and a cell preservative. After blood collection, the cells expressing cluster of differentiation (CD) 146 are immunomagnetically captured using ferrofluids coated with anti-CD146 monoclonal antibodies. The enriched cells are then labeled with 4′,6-diamidino-2-phenylindole, CD105 and CD45. CECs were defined when its morphological features are consistent with that of a cell and it exhibits the correct phenotypes (positive for CD146, CD105 and 4′,6-diamidino-2-phenylindole, negative for hematopoietic marker CD45). Results are reported as the number of CECs per 4.0 mL of blood.

## Results

### Baseline characteristics

A total of 47 patients [28 males and 19 females (59.6%); median age, 61 years; age range, 39–73 years] were enrolled from **7** centers across Japan from October 2010 to April 2012. Patient demographics and disease characteristics are summarized in Table 1. All patients presented with adenocarcinoma, and 39 patients (83.0%) had stage-IV disease. The ECOG-PS score was 0 in 31 patients (66.0%) and 1 in 16 patients (34.0%). EGFR mutations were detected in 13 patients (27.7%).

**Table 1:** Baseline characteristics of the study population

	*N* = 47
Age	61 (39–73)
Gender (male/female)	28/19
Smoking history	33 (70.2%)
ECOG Performance Status	31/16
0	31 (66.0%)
1	16 (34.0%)
Histology	
adenocarcinoma	47 (100%)
Staging	
IIIB	5 (10.6%)
IV	39 (83.0%)
recurrent	3 (6.4%)
EGFR mutation	
wild type	32 (68.1%)
exon19 deletion	7 (15.2%)
exon21 L858R	5 (10.9%)
exon21 L858R + de novo T790 M	1 (2.1%)
unknown	2 (4.2%)
Prior treatment	
surgery	3 (6.4%)
palliative radiotherapy	3 (6.4%)
EGFR tyrosine kinase inhibitor	0

Abbreviations: *ECOG* eastern cooperative oncology group, *EGFR* epidermal growth factor receptor

### Treatment delivery and efficacy

**Fig. 1:**
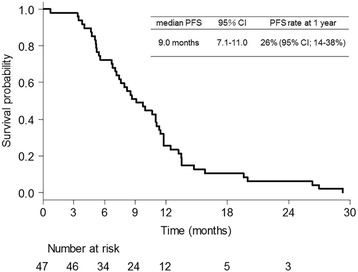
Kaplan–Meier curve for progression-free survival

**Fig. 2:**
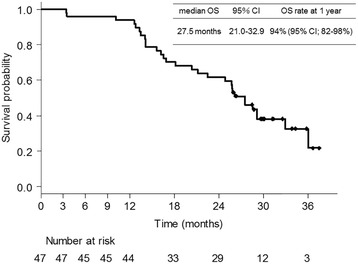
Kaplan–Meier curve for overall survival

A total of 44 patients (93.6%) received 4 cycles of cisplatin, docetaxel, and bevacizumab, and 41 patients (87.2%) received ≥1 cycle of maintenance bevacizumab. The median number of total treatment cycles was 9 (range: 1–21). Cisplatin dose reduction was required in 13 patients (27.7%), and docetaxel dose reduction was required in 12 patients (25.5%). A total of 35 patients (74.5%) achieved partial responses with an ORR of 74.5% [95% confidence interval (CI): 59.7–86.1%]. Eleven patients (23.4%) archived a stable disease status with a disease control rate of 97.9% (95% CI: 88.7–99.9%). Median PFS was 9.0 (95% CI: 7.0–11.3) months (Fig. 1). PFS rate at 1 year was 26% (95% CI: 14–38%). Median OS was 27.5 (95% CI: 21.1–32.9) months (Fig. 2). OS rate at 1 year was 94% (95% CI: 82–98%).

### Adverse events (AEs)

Hematological and non-hematological toxicities of all patients are summarized in Table 2. The most common grade ≥ 3 AE in the induction therapy was neutropenia (95.7%), followed by leukopenia (59.6%), hypertension (31.9%), anorexia (12.8%), nausea (12.8%), and febrile neutropenia (8.5%). One patient (2.1%) developed alveolar hemorrhage (Grade 5) after 4 cycles of induction therapy [15]. During the maintenance therapy of bevacizumab (*N* = 41), the grade ≥ 3 AE was rarely observed, except for hypertension (34.1%).

**Table 2:** Adverse events during induction and maintenance phases

	Induction phase: 1–4 cycles (*N* = 47)		Maintenance phase: 5 cycles- (*N* = 41)	
	All grade	Grade ≥ 3	All grade	Grade ≥ 3
Leukopenia	46 (97.8%)	28 (59.6%)	4 (9.8%)	0
Neutropenia	46 (97.8%)	45 (95.7%)	2 (4.9%)	0
Febrile neutropenia	4 (8.5%)	4 (8.5%)	0	0
Anemia	47 (100%)	2 (4.3%)	27 (65.9%)	0
Thrombocytopenia	27 (57.4%)	0	7 (17.1%)	0
Hypoalbuminemia	43 (91.5%)	0	7 (17.1%)	0
AST increased	12 (25.5%)	0	7 (17.1%)	0
ALT increased	17 (36.2%)	1 (2.1%)	5 (12.2%)	0
ALP increased	18 (37.3%)	0	8 (19.5%)	0
Creatinine increased	25 (53.2%)	0	17 (41.5%)	0
Anorexia	43 (91.5%)	6 (12.8%)	3 (7.3%)	0
Nausea	39 (83.0%)	6 (12.8%)	4 (9.8%)	0
Vomiting	13 (27.7%)	0	0	0
Diarrhea	17 (36.2%)	3 (6.4%)	2 (4.9%)	0
Constipation	19 (40.4%)	0	2 (4.9%)	0
Weight loss	27 (57.4%)	0	8 (19.5%)	0
Fatigue	24 (51.1%)	0	6 (14.6%)	0
Alopecia	38 (80.9%)	0	19 (46.3%)	0
Oral mucositis	10 (21.3%)	0	1 (2.4%)	0
Sensory neuropathy	8 (17.0%)	0	10 (24.4%)	0
Fever	7 (14.9%)	1 (2.1%)	2 (4.9%)	0
Hypertension	44 (93.6%)	15 (31.9%)	34 (82.9%)	14 (34.1%)
Proteinuria	32 (68.1%)	0	13 (31.7%)	0
Nasal bleeding	9 (19.1%)	0	5 (12.2%)	0

Abbreviations: *AST* aspartate aminotransferase, *ALT* alanine aminotransferase, *ALP* alkaline phosphatase

### Analysis of CECs

**Fig. 3:**
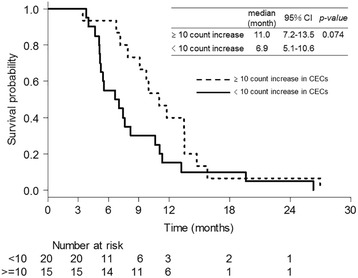
Comparison of progression-free survival based on the change in circulating endothelial cell counts from baseline to day 8

In 35 patients, CEC count was measured on days 1 and 8 (Additional file 1: Figure S1; Additional file 2: Table S1). CECs increased to ≥10 count in 15 patients (42.9%) from the baseline to day 8. Survival curves were compared on the basis of the change from baseline to day 8 (Fig. 3). PFS was longer in patients with ≥10 count increase in CECs than in patients with < 10 count increase in CECs (respective median PFS of 11.0 months and 6.90 months, respectively) although the difference was not statistically significant (*p = 0.074*).

## Discussion

The present study demonstrated three important clinical observations. First, the combined therapy of bevacizumab, cisplatin, and docetaxel, followed by bevacizumab maintenance treatment revealed high efficacy in patients with advanced non-squamous NSCLC. Second, a high frequency of grade ≥ 3 neutropenia and leukopenia were observed although the other AEs were almost manageable. Third, PFS was longer in patients with ≥10 count increase in CECs than in patients with < 10 count increase in CECs.

The past two single-arm phase II studies of bevacizumab (15 mg/kg) in combination with docetaxel (75 mg/m^2^) and cisplatin (75 or 80 mg/m^2^) as the first-line treatment of patients with metastatic non-squamous NSCLC have revealed favorable anti-tumor effect, with an ORR of 33.3–63.0%, a median PFS of 4.4–7.8 months, and a median OS of 13.3–13.5 months [11, 12]. Despite the use of Japanese reduced dose of docetaxel (60 mg/m^2^), the present study revealed much higher ORR and much longer PFS and OS than in the above-mentioned past two studies and the past studies using other third generation chemotherapy plus platinum with bevacizumab, such as E4599, AVAiL, AVAPERL and PointBreak trials [7, 8, 16, 17]. These results suggest that the anti-tumor effect of cisplatin, docetaxel, and bevacizumab combination therapy is extremely promising. Furthermore, these results also suggested that the present regimen may be more effective for Japanese patients. When comparing global phase 3 study (E4599 trial) and Japanese phase 2 study (JO19907 trial) of carboplatin, paclitaxel and bevacizumab combination therapy, Japanese trial revealed higher ORR and longer PFS than global trial [7, 18]. Similarly, when comparing global phase 3 study (REVEL trial) and Japanese phase 2 bridging study (JVCG trial) of docetaxel plus ramucirumab, a monoclonal antibody that inhibits VEGFR-2, Japanese trial revealed higher ORR and longer PFS than global trial despite Japanese reduced dose of docetaxel [19, 20]. It was speculated that there may be an ethnic difference in the efficacy of taxanes and angiogenesis inhibitors combination therapy.

In terms of toxicity, neutropenia and leukopenia were the most common AEs among grade ≥ 3 AEs in the past phase II studies of cisplatin, docetaxel, and bevacizumab (grade ≥ 3 neutropenia occurred in 18.7–22.0% patients and grade ≥ 3 leukopenia in 8.4–9.8% patients, respectively) [11, 12]. However, in the present study, the incidence of neutropenia and leukopenia was higher than in the past two studies, despite the use of Japanese reduced dose of docetaxel (60 mg/m^2^). According to the past phase III trials of docetaxel monotherapy, grade ≥ 3 neutropenia was more frequently observed in Japanese patients treated with 60 mg/m^2^ (73.3–85.9%) than in the patients from the Western countries treated with 75 mg/m^2^ dose (21–37%) although the efficacy was almost equivalent in both the cases [21–29]. The results of the phase III trials with docetaxel (60 mg/m^2^) plus cisplatin in Japanese patients with advanced NSCLC have revealed that the incidence of grade ≥ 3 neutropenia and leukopenia was 73.4–74.2% and 45.7–55.2%, respectively [30, 31]. Moreover, randomized phase II trial of docetaxel versus docetaxel plus bevacizumab in Japanese patients with NSCLC, who were previously treated with bevacizumab plus a platinum-based doublet, has demonstrated that additional bevacizumab increased the incidence of grade ≥ 3 leukopenia and neutropenia [32]. These results suggest that severe neutropenia and leukopenia are the most serious AEs of the present regimen. Nevertheless, in this study, non-hematological toxicities were almost manageable, the transfer rate to the bevacizumab maintenance therapy was high, and the number of treatment cycles was more than in other studies. The prophylactic use of pegylated granulocyte colony-stimulating factor ensures the higher safety of this promising regimen.

To date, no useful biomarker has been established for predicting the efficacy of bevacizumab. CECs are mature endothelial cells that are sloughed from the vessel wall, and increase in CECs in the peripheral blood is expected to serve as a potential biomarker for predicting response to bevacizumab [14, 33–35]. Because the CEC counts appear to vary according to the type of cancer or the types of assay methods, the usefulness of CECs remains controversial. However, several studies have suggested that change in the CEC count after chemotherapy serves as a predictive biomarker for the effect of chemotherapy, particularly for bevacizumab combination therapy. Calleri et al. [14] have reported that tumor progression under bevacizumab-combined chemotherapy was associated with a significant CEC count decrease in advanced breast cancer patients [14]. In addition, Bidard et al. [36] have reported that in breast cancer patients receiving bevacizumab combination with taxane-based therapy, increase in the CEC count during treatment was associated with improved time to disease progression, whereas the baseline CEC counts were not associated with time to progression. CECs are thought to represent an indirect marker of vascular remodeling and turnover [37], thus, it was speculated that increase in CEC reflects the degree of regression or normalization of existing tumor vasculature and the inhibition of new and recurrent tumor vessel growth by bevacizumab. Regarding patients with advanced NSCLC, there has been no prospective study of a bevacizumab-containing regimen that evaluated the correlation between the change in CEC count and treatment efficacy. Therefore, it is important to accumulate more cases from a number of hospitals to further validate the findings of the present study.

This study had some limitations. First, the lack of a standard assay for CEC counting may hinder the clinical application of the proposed concept in clinical practice. However, the variability in CEC values is smaller among studies that measure CECs by CellSearch® than those that use other systems. Second, 11 of 13 patients with EGFR-sensitive mutations received EGFR-tyrosine kinase inhibitor as a second- or third-line treatment, which warrants a careful interpretation of the evaluated OS values.

## Conclusions

Bevacizumab, cisplatin, and docetaxel in combination, followed by bevacizumab alone as a maintenance treatment was highly effective in patients with non-squamous NSCLC despite the high incidence of grade 3/4 neutropenia. The increases in CECs count between days 1 and 8 may predict the efficacy of our bevacizumab-contained treatment regimen.

## Additional files

Additional file 1: **Figure S1**. Trial profile. Abbreviations: CEC, circulating endothelial cell. (JPEG 147 kb)

Additional file 2: **Table S1**. CEC data and treatment efficacy of 35 patients whose CEC count was measured on days 1 and 8. Abbreviations: EGFR, epidermal growth factor receptor; CEC, circulating endothelial cell; PR, partial response; SD, stable disease; PFS, progression-free survival; OS, overall survival. (DOCX 22 kb)

## Abbreviations

AEs: Adverse events
ALT: Alanine aminotransferase
AST: Aspartate aminotransferase
CD: Cluster of differentiation
CEC: Circulating endothelial cell
CI: Confidence interval
CT: Computed tomography
CTCAE: Common terminology criteria for adverse events
ECOG: Eastern cooperative oncology group
EGFR: Epidermal growth factor receptor
MRI: Magnetic resonance imaging
NSCLC: Non-small cell lung cancer
ORR: Objective response rates
PFS: Progression-free survival
PR: Partial response
PS: Performance status
RECIST: Response evaluation criteria
TKI: Tyrosine kinase inhibitor
TORG: Thoracic oncology research group
VEGF: Vascular endothelial growth factor
