# Supplementary figures and images for: Phase II study of bevacizumab, cisplatin, and docetaxel plus maintenance bevacizumab as first-line treatment for patients with advanced non-squamous non-small-cell lung cancer combined with exploratory analysis of circulating endothelial cells: Thoracic Oncology Research Group (TORG)1016
Source: BMC Cancer. 2018 Mar 2;18:241. doi: 10.1186/s12885-018-4150-y (PMC5833040; doi:10.1186/s12885-018-4150-y)

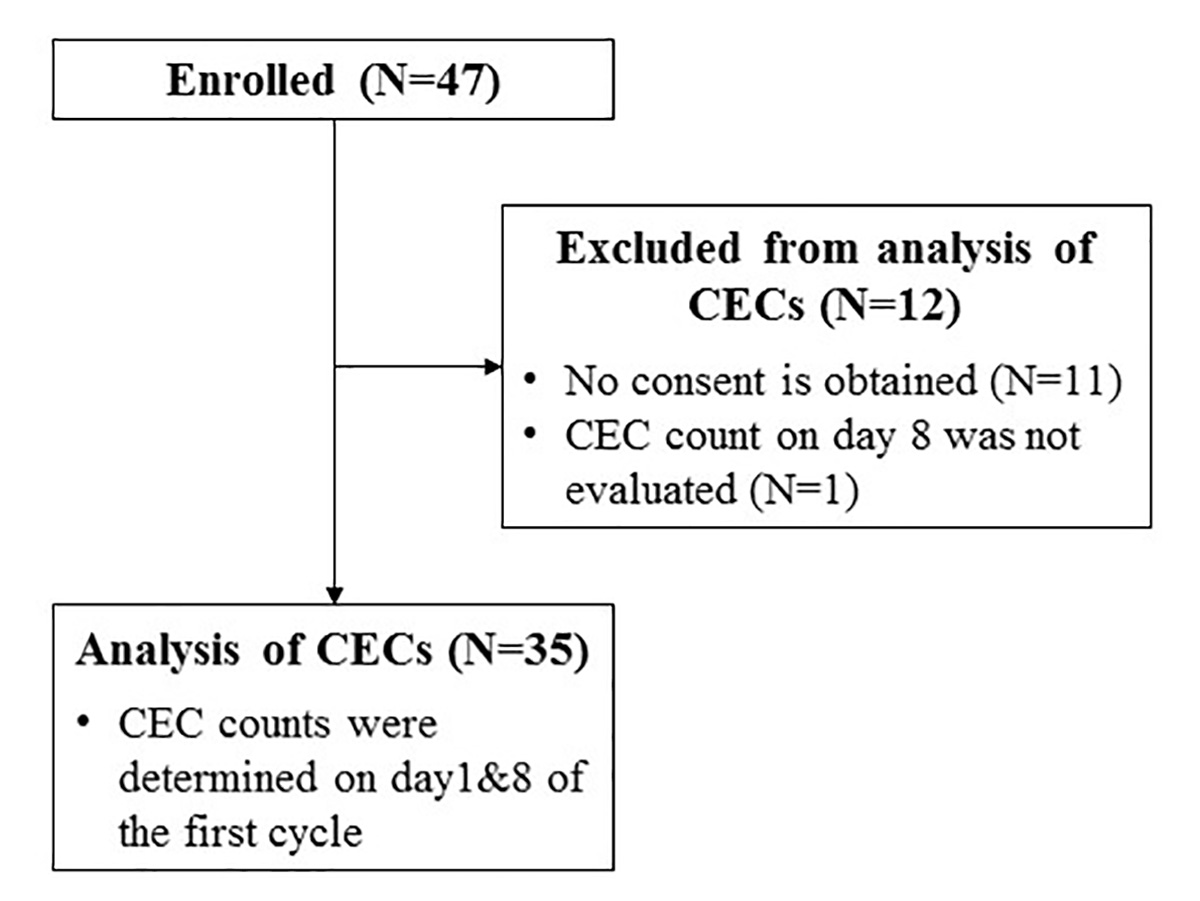

Supplement: Supplementary file 1 — Figure S1. Trial profile. Abbreviations: CEC, circulating endothelial cell. (JPEG 147 kb) [file 12885_2018_4150_MOESM1_ESM.jpg]
