# Supplementary material for: Phase II study of bevacizumab, cisplatin, and docetaxel plus maintenance bevacizumab as first-line treatment for patients with advanced non-squamous non-small-cell lung cancer combined with exploratory analysis of circulating endothelial cells: Thoracic Oncology Research Group (TORG)1016
Source: BMC Cancer. 2018 Mar 2;18:241. doi: 10.1186/s12885-018-4150-y (PMC5833040; doi:10.1186/s12885-018-4150-y)
Supplement: Supplementary file 2 — Table S1. CEC data and treatment efficacy of 35 patients whose CEC count was measured on days 1 and 8. Abbreviations: EGFR, epidermal growth factor receptor; CEC, circulating endothelial cell; PR, partial response; SD, stable disease; PFS, progression-free survival; OS, overall survival. (DOCX 22 kb) [file 12885_2018_4150_MOESM2_ESM.docx]

**Table S1. CEC data and treatment efficacy of 35 patients whose CEC count was measured on days 1 and 8.**

| Case | EGFR mutation | CEC count | | | Best response | PFS (month) | OS (month) |
| --- | --- | --- | --- | --- | --- | --- | --- |
|  |  | day1 | day8 | ΔCEC |  |  |  |
| 1 | wild type | 18 | 15 | -3 | PR | 11.3 | 29.1 |
| 2 | wild type | 13 | 31 | 18 | PR | 11.8 | 25.8 |
| 3 | wild type | 14 | 39 | 25 | PR | 13.6 | 33.0 |
| 4 | exon19 del | 101 | 220 | 119 | PR | 9.1 | 13.4 |
| 5 | wild type | 12 | 15 | 3 | PR | 5.1 | 14.1 |
| 6 | wild type | 88 | 87 | -1 | PR | 11.1 | 26.1 |
| 7 | exon21 ins | 36 | 174 | 138 | SD | 3.5 | 3.5 |
| 8 | wild type | 52 | 95 | 43 | SD | 7.2 | 37.5 |
| 9 | exon19 del | 30 | 28 | -2 | SD | 5.2 | 27.5 |
| 10 | wild type | 27 | 29 | 2 | PR | 10.6 | 29.1 |
| 11 | wild type | 107 | 22 | -85 | PR | 5.5 | 35.8 |
| 12 | wild type | 13 | 13 | 0 | PR | 6.7 | 25.8 |
| 13 | exon21 ins | 22 | 23 | 1 | PR | 8.2 | 22.5 |
| 14 | wild type | 162 | 91 | -71 | PR | 7.6 | 36.6 |
| 15 | wild type | 233 | 317 | 84 | PR | 8.0 | 21.2 |
| 16 | wild type | 10 | 27 | 17 | SD | 9.7 | 34.3 |
| 17 | wild type | 60 | 84 | 24 | PR | 6.8 | 10.1 |
| 18 | wild type | 102 | 43 | -59 | PR | 5.2 | 13.3 |
| 19 | exon19 del | 170 | 24 | -146 | SD | 3.8 | 16.5 |
| 20 | wild type | 48 | 56 | 8 | SD | 4.7 | 14.0 |
| 21 | wild type | 149 | 84 | -65 | PR | 7.5 | 16.9 |
| 22 | wild type | 207 | 222 | 15 | PR | 11.0 | 11.7 |
| 23 | exon19 del | 11 | 185 | 174 | PR | 14.8 | 30.1 |
| 24 | wild type | 188 | 33 | -155 | PR | 19.6 | 31.3 |
| 25 | wild type | 11 | 16 | 5 | PR | 7.1 | 28.6 |
| 26 | exon21 ins | 18 | 43 | 25 | PR | 13.5 | 29.8 |
| 27 | wild type | 20 | 43 | 23 | PR | 13.5 | 18.2 |
| 28 | wild type | 8 | 47 | 39 | PR | 15.8 | 28.4 |
| 29 | wild type | 185 | 91 | -94 | PR | 13.2 | 29.6 |
| 30 | exon21 ins | 60 | 22 | -38 | PR | 5.6 | 28.7 |
| 31 | wild type | 29 | 14 | -15 | SD | 5.1 | 12.8 |
| 32 | exon19 del | 12 | 12 | 0 | PR | 26.3 | 26.3 |
| 33 | wild type | 18 | 68 | 50 | PR | 10.0 | 26.2 |
| 34 | wild type | 12 | 23 | 11 | PR | 26.9 | 25.9 |
| 35 | wild type | 32 | 23 | -9 | PR | 4.0 | 14.1 |

Abbreviations: EGFR, epidermal growth factor receptor; CEC, circulating endothelial cell; PR, partial response; SD, stable disease; PFS, progression-free survival; OS, overall survival
